# The effect of maternal and child health resource allocation on efficiency in China

**DOI:** 10.1038/s41598-026-54802-8

**Published:** 2026-05-26

**Authors:** Zhaoyang Wang, Xinyue Sun, Zhen Wang, Yuxin Wang, Lu Cheng, Jiaxin Xie, Kai Meng

**Affiliations:** 1 School of Public Health, Capital Medical University, No.10 Xitoutiao, Youanmenwai Street, Fengtai District, Beijing, 100069 China; 2 Beijing Tiantan Hospital, Capital Medical University, No. 119 South Fourth Ring West Road, Fengtai District, Beijing, 100070 China

**Keywords:** Maternal and child health resources, Efficiency, Integrated health resource density index, Three-stage DEA model, Spatial econometric model, Development studies, Environmental social sciences, Health care, Social policy

## Abstract

Maternal and child healthcare services constitute a vital component of the healthcare system and play a crucial role in safeguarding population health. However, in China, challenges such as the irrational allocation of MCH resources and suboptimal operational efficiency remain prevalent. This study aims to investigate the impact of resource allocation on the efficiency of the maternal and child healthcare system in China. Data for 31 provinces and municipalities in China were obtained from the China Health Statistical Yearbook and the China Statistical Yearbook covering the period 2017 to 2021. The comprehensive Maternal and Child Health resource density index was developed by applying the entropy weight method to analyze the allocation of MCH resources across provinces. Furthermore, a three-stage Data Envelopment Analysis (DEA) model was employed to calculate the efficiency of the provincial MCH systems. Finally, a spatial Durbin model was applied to examine the effect of the comprehensive MCH resource density on system efficiency. Between 2017 and 2021, the comprehensive Maternal and Child Health (MCH) resource density index in most regions of China increased from 0.108 to 0.138. In contrast, the average technical efficiency declined from 0.943 to 0.920, with substantial disparities observed across regions. The Moran’s I index for MCH resource efficiency ranged from − 0.349 to − 0.245, indicating significant spatial autocorrelation. Key factors influencing the technical efficiency of MCH resources included the comprehensive CHRDI in maternal and child health, urbanization rate, and total health expenditure per capita (*P* < 0.05). While the allocation of maternal and child health resources in China improved between 2017 and 2021, significant regional disparities persist. The study further reveals that the level of MCH resource allocation exerts spatial spillover effects on efficiency. Based on the current data trend, optimizing resource allocation may require a combination of policy guidance, financial input, performance evaluation, regional collaboration, and other coordination strategies.

## Background

The health and living conditions of women and children are widely regarded as a vital sign of national development and societal standing^1^. As a fundamental safeguard for the health rights of these populations, maternal and child health (MCH) resources represent a crucial element of the public health system^2^. The global importance of this issue was formally acknowledged at the United Nations Millennium Summit in 2000, which emphasized maternal and child health as a key international priority.

According to data from China’s Seventh National Census, children under the age of 14 account for 17.95% of the total population, while women represent 48.76%, collectively constituting approximately two-thirds of the national total^3^. This demographic structure underscores the significant proportion of the population comprised by women and children. At the policy level, the Chinese government has accorded high priority to maternal and child health (MCH). The Outline of “Healthy China 2030” identifies women and children as key target groups and emphasizes the need to enhance their health status. Furthermore, a series of strategic documents-including the Program for the Development of Chinese Women (2021–2030), the Program for the Development of Chinese Children (2021–2030), and the corresponding implementation plan issued by the National Health Commission—have been promulgated to promote the high-quality development of MCH services and elevate the standard of care^4^. Additionally, the Measures for Performance Assessment of Maternal and Child Health Institutions aim to enhance service capacity and operational management through rigorous evaluation mechanisms. These multi-faceted policy efforts reflect China’s comprehensive support for MCH development, establishing it as a critical domain within the nation’s public health agenda.

In recent years, China has progressively entered an aging society, leading to growing pressures on the pension system and imposing substantial burdens on single-child families. To mitigate the social challenges associated with population aging, the Chinese government has actively and sequentially adjusted its population policies. Initially transitioning from the earlier “one-child policy” to the “selective two-child policy” in 2013, it further introduced the “universal two-child policy” in 2016, and most recently, the “three-child policy” in 2021. These shifts reflect rising societal demand for fertility and have been accompanied by an increasing proportion of advanced maternal age pregnancies. This trend not only raises the need for maternal and child health resources but also elevates the quality requirements for MCH service delivery^5^. Against this backdrop, China’s MCH sector is confronting new challenges, presenting both opportunities and pressures for the development of MCH institutions.

Existing research indicates that although maternal and child health (MCH) resources in China have been increasing annually, several prominent issues persist. These include a structural imbalance between the growing demand for and the supply of MCH services, with the total volume of services remaining relatively insufficient to meet population needs^6^. In terms of infrastructure, many MCH institutions face inadequacies in hospital beds and supporting medical equipment^7^. There is also a shortage of human resources in the MCH sector, and the equity of workforce distribution across institutions requires improvement^8^. Moreover, the allocation efficiency of MCH resources is generally low, leading to a coexistence of resource shortages and wastage, wherein existing resources are not fully utilized^9^. These challenges have considerably impeded the healthy and sustainable development of MCH services in China.

Furthermore, international comparisons reveal a significant gap between China and developed countries in the availability of MCH resources^8,10^. Although both central and local governments have continuously increased investment in MCH in recent years-leading to notable improvements in maternal and child health outcomes—issues such as inefficiency and inequity in MCH service delivery remain long-standing. The MCH service network continues to lag within the broader healthcare system^11,12^. The rational allocation and effective utilization of MCH resources are essential for enhancing the health level of the entire population and achieving the strategic goals of Healthy China. They are also critical to the health quality of China’s future population and its reserve force for social development.

Existing studies have identified multiple factors influencing the allocation efficiency of maternal and child health (MCH) resources, including population density, economic development level, government support, medical equipment investment, financial input, and the quality structure of healthcare talent^13,14^. This suggests that the allocation level of MCH resources may, to some extent, affect institutional efficiency. Furthermore, relevant research has confirmed that continuously improving the rationality of MCH resource allocation exerts a positive impact on the operational efficiency of MCH institutions^15^.

While some studies have separately examined the equity and efficiency of health resource allocation, few have systematically analyzed the correlation between these two dimensions^16^. As an integral component of health resources, both the allocation status and operational efficiency of MCH resources serve as critical indicators for evaluating service supply, and their interrelationship is likely to be complex. Investigating this relationship would help address a notable gap in the current literature and provide an evidence-based foundation for policymaking. Clarifying this linkage would also contribute to safeguarding the health rights and interests of women and children, improving the quality of MCH services, and promoting the overall development of MCH systems. Therefore, this study aims to analyze both the allocation of MCH resources and the efficiency of MCH institutions, with a specific focus on exploring the impact of MCH resource allocation levels on institutional operational efficiency.

## Materials and methods

### Data sources

This study selected maternal and child healthcare (MCH) institutions across 31 provincial-level administrative regions in mainland China (excluding Hong Kong, Macao, and Taiwan) as the research subjects. In China, MCH institutions are defined as independent public health entities primarily responsible for providing healthcare services to women and children, in accordance with the Measures for the Administration of Maternal and Child Health Institutions. Unlike general or obstetrics and gynecology hospitals, which focus predominantly on clinical treatment, MCH institutions emphasize preventive and systematic healthcare services. Therefore, this study excludes general hospitals and specialized obstetrics and gynecology hospitals. Based on data availability and representativeness, input and output variables for evaluating the efficiency of MCH services were collected from the China Health Statistical Yearbook and the China Statistical Yearbook for the period 2017–2021. These variables included the number of MCH institutions, health technicians, and beds, as well as the number of live births, newborn visit rate, health management rate of children under 7 years old, system management rate, and premarital examination rate. Control variables such as population density, per capita total health expenditure, and urbanization rate were also obtained from the same sources. Using these data, this study assesses the allocation level and service efficiency of MCH resources in China and further examines the relationship between the two.

### Comprehensive health resource density index (CHRDI)

The Health Resource Density Index (HRDI) is a composite indicator that integrates both population and geographical dimensions to reflect the concentration of health resources. By incorporating these two factors, the HRDI mitigates potential bias arising from reliance on a single demographic or geographic metric, thereby offering a more balanced assessment of the comprehensive distribution level of health resources across regions^17^. It is calculated as the square root of the product of health resources per thousand population and health resources per square kilometer, as follows:

$$HRDI = \sqrt {\frac{{\text{Quantity of health resources}}}{{\text{Thousand people}}} \times \frac{{\text{Quantity of health resources}}}{{\text{Square kilometer}}}}$$

The Comprehensive Health Resource Density Index (CHRDI) is a multidimensional metric constructed by assigning weights to individual health resource indicators using the entropy weight method^18^. In accordance with the principle of “multiplying within the same dimension and summing across different dimensions,” the CHRDI is calculated as the weighted sum of the products of each indicator’s assigned weight and its corresponding Health Resource Density Index (HRDI) value. Specifically, it is expressed as:$CHRDI = \sum\nolimits_{i = 1}^{{\mathrm{m}}} {HRDI \times W_{j} }$. The greater the CHRDI value, the greater the density of maternal and child health care resources and the higher the allocation level.

Following the variable selection criteria established in prior research^19^, this study first calculated the maternal and child health (MCH) resource density index across three dimensions—number of institutions, number of health technicians, and number of beds—for China from 2017 to 2021. Subsequently, the entropy weight method was applied to determine the weight of each indicator. By integrating these weights, the comprehensive health resource density index (CHRDI) for MCH resources was derived to provide a multidimensional and accurate reflection of MCH resource allocation within China’s specialized healthcare institutions.

The constituent indicators of the comprehensive maternal and child health resource density index are grounded in the theory of health resource allocation. This study constructs the index across three core dimensions—institutions, human resources, and beds—to comprehensively reflect the physical allocation level of maternal and child health resources in each region. The number of institutions refers to the count of specialized maternal and child health institutions in each region, reflecting the accessibility and geographical coverage density of maternal and child health services and serving as the spatial carrier of resource allocation. The number of health technicians refers to the total number of personnel engaged in health technical work within maternal and child health institutions, indicating the level of human capital investment and representing the core element of service supply capacity. The number of beds refers to the actual number of beds in maternal and child health institutions, reflecting inpatient service capacity and resource reserve levels. These three indicators are widely applied in health resource density evaluation studies.

### The entropy method

Entropy weight method is an objective valuation method based on information entropy theory. It determines the weight of indicators in comprehensive evaluation by calculating the amount of information contained in each indicator, which can objectively reflect the proportion of each indicator in the entire evaluation system^20^. The size of X_ij_ value and weight W_j_ value represent the importance of this index in comprehensive evaluation. This study uses this method to select the indicators of the number of maternal and child health institutions, health technicians and number of beds in each province to calculate the comprehensive maternal and child health resource density index in each province. The calculation process is decomposed into the following five steps: First, the index is standardized by the extreme value standardization method, that is, when X_ij_ is a positive indicator, the standardized X_ij_ is the ratio range of X_ij_ and the minimum (Formula 1), and when X_ij_ is a negative indicator, the standardized X_ij_ is the ratio range of the maximum and Xij (Formula 2). In the second step, the specific gravity value of the index of the j item i region P_ij_ (Formula 3) is calculated, that is, the ratio of X_ij_ and the sum of X_ij_; Secondly, the entropy value e_j_ of the JTH index is calculated (Formula 4). The fourth step is to obtain the difference coefficient of the J-th index (Formula 5); Finally, the weight W_j_ of the J-th index is calculated, that is, the ratio of g_i_ to the sum of g_i_ (Formula 6).

1 $$X_{ij}^{\prime } = \frac{{X_{ij} - X_{\min (j)} }}{{X_{\max (j)} - X_{\min (j)} }}$$

2 $$X_{{{\mathrm{ij}}}}{\prime} = \frac{{X_{{{\mathrm{max}}(j)}} - X_{ij} }}{{X_{\max (j)} - X_{\min (j)} }}$$

3 $$P_{ij} = \frac{{X_{ij}{\prime} }}{{\sum\nolimits_{i = 1}^{m} {X_{ij}{\prime} } }}$$

4 $$e_{j} = - \frac{1}{\ln m}\sum\nolimits_{i = 1}^{{\mathrm{m}}} {P_{ij} \times \ln P_{ij}^{{}} }$$

5 $${\mathrm{g}}_{i} = 1 - e_{j}$$

6 $$W_{j} = \frac{{g_{i} }}{{\sum\nolimits_{i = 1}^{m} {g_{i} } }}$$

### Three-stage DEA model

Data Envelopment Analysis (DEA) is a non-parametric method for evaluating the relative efficiency of decision-making units (DMUs) with multiple inputs and outputs. By employing linear programming techniques, DEA assesses the operational efficiency of comparable units and has been widely applied in studies of system performance^21^. The conventional DEA model, initially proposed by Charnes et al.^22^, has certain limitations: it fails to distinguish the influence of external environmental factors and random noise, attributing all deviations to managerial inefficiency^17^, and is not well-suited for situations involving variable returns to scale^23^. While some scholars, such as Ibrahim MD have applied the traditional DEA model to analyze the technical efficiency of maternal and child health (MCH) resources^24^, others like Herwartz H have employed stochastic frontier analysis (SFA) to evaluate the allocation efficiency of healthcare resources^25^. To address the limitations of conventional DEA and produce more robust results, this study adopts an integrated three-stage DEA approach, which incorporates controls for environmental factors and statistical noise. The analytical procedure consists of the following three stages: First, the traditional DEA model was adopted, and Deap2.1 software was used to calculate the allocation efficiency and input relaxation variables of maternal and child health resources in each province; then, Frontier4.1 software was used to conduct stochastic frontier analysis. Relaxation variables were put in as dependent variables and external environment factors as independent variables, and an SFA model was established to obtain the adjusted input values so as to eliminate the influence of environmental factors and random errors^26^. Finally, the traditional DEA model is used again, the adjusted input value is replaced by the original value, and the adjustment efficiency of the provincial and municipal maternal and child health care system is recalculated to make the results more real and reliable.

Comprehensive technical efficiency (TE) serves as an integrated metric for evaluating both the resource allocation capacity and utilization effectiveness of decision-making units (DMUs). In this study, TE values derived from the Data Envelopment Analysis (DEA) model-which range between 0 and 1, with higher values indicating greater relative efficiency-are used to assess maternal and child health (MCH) system performance^27^. Input slack variables, representing gaps between actual and optimal input levels, further reveal inefficiencies in resource use. Guided by the requisite causal relationship between inputs and outputs in DEA modeling^28^, this study selects capital inputs (number of institutions and beds) and human resources (number of health technicians) as input indicators. Outputs include service coverage and demand measures: newborn visit rate (child health service), system management rate (maternal health management), premarital check-up rate (pre-pregnancy care), health management rate of children under 7 years (continued child health monitoring), and number of live births (scale of MCH demand)^29^. Environmental variables-per capita GDP, population density, and per capita total health expenditure-are incorporated to control for contextual influences^19^, ensuring a robust and interpretable evaluation of MCH resource efficiency.

Output indicators of maternal and child health system efficiency: This study adopts the “input–output” analysis framework for evaluating health system efficiency and incorporates service volume, service quality, and coverage level when selecting output indicators. The number of live births refers to the total number of live births in a given region within one year. It reflects the basic service output of the maternal and child health care system and serves as a direct indicator for measuring the system’s operational load and output scale. The neonatal visit rate is defined as the proportion of newborns receiving postnatal visits relative to the number of live births during the same period. This indicator reflects the accessibility and timeliness of early neonatal health care services. The health care management rate for children under 7 years old refers to the proportion of children in this age group who receive systematic health care management. It indicates the breadth of coverage of child health services. The systematic management rate refers to the proportion of pregnant women who receive systematic prenatal examinations, hospital delivery, and postnatal visits within 42 days from pregnancy to the postpartum period. This indicator reflects the continuity and comprehensiveness of maternal health care services. The premarital examination rate refers to the proportion of individuals receiving premarital medical examinations relative to the number of registered marriages. It reflects the coverage level of preconception health care and primary prevention services for birth defects. Together, these five indicators constitute the core performance measures of maternal and child health care.

### Moran index

Spatial autocorrelation is an important method of quantitative analysis in geography, and its correlation index can be used to judge the spatial correlation of health service resource allocation in a given region. Moran’s Index is a statistical index used to evaluate whether data has spatial dependence and spatial autocorrelation from the geographical dimension. It is mainly used to judge whether there is a correlation between things reflected in data within a certain range, which can be divided into the global Moran index and the local Moran index^30^. In this study, the global Moran Index and local Moran Index were used to measure the spatial aggregation of maternal and child health resource allocative efficiency in China during 2017–2021. Among them, the Global Moran index IG (global Moran’s I) is an indicator reflecting the overall spatial autocorrelation of maternal and child health allocative efficiency. It can be used to analyze the overall spatial aggregation degree of the allocative efficiency of maternal and child health care in China. The value of IG ranges from -1 to 1, where IG greater than 0 indicates positive spatial autocorrelation, allocative efficiency of maternal and child health care presents aggregation characteristics in spatial distribution, IG less than 0 indicates negative spatial autocorrelation, and IG approaching 0 indicates no obvious spatial correlation. Without spatial autocorrelation, the larger the absolute value of Moran’s I is, the stronger the spatial correlation is^31^. Local Moran Index IL (local Moran’s I) is a further analysis of the spatial autocorrelation between adjacent subregions in the overall MCH allocative efficiency reflected by the global Moran Index. It belongs to the spatial decomposition of the global Moran Index and is used to find out the specific parts of the overall MCH allocative efficiency that are clustered^32^. It can be used to analyze the spatial correlation degree between the allocative efficiency of maternal and child health care in China’s provinces and neighboring regions and further clarify the spatial aggregation state and local characteristic differences of the allocative efficiency of maternal and child health care in China.

In order to clarify the specific distribution of spatial aggregation, according to the degree of spatial aggregation calculated by the Moran index, the spatial distribution of each space and its adjacent areas can be divided into four types: This region and adjacent regions are “H–H-type aggregation” with high aggregation; this region and adjacent regions are “L-L-type aggregation” with low aggregation; this region is “L–H-type aggregation” with low aggregation, but adjacent regions are “L–H-type aggregation” with high aggregation, but adjacent regions are^33^. In this study, Moran’s I model in Stata17 software was used to analyze the spatial autocorrelation of technical efficiency values of maternal and child health resources in 31 provinces of China, and *P* < 0.05 was considered statistically significant.

### Spatial econometric model

When efficiency exhibits significant spatial correlation, it is necessary to employ spatial econometric models to investigate its influencing factors. Common spatial econometric models include the spatial lag model (SLM), the spatial error model (SEM), and the spatial Durbin model (SDM). In this study, the selection of control variables for the spatial econometric model took into account both the socioeconomic status and health system characteristics of each region. Specifically, the following variables were included: urbanization rate, population density and total health expenditure per capita, number of health technicians, number of institutions, and number of live births^34,35^. To further examine the spatial dependence between the dependent and independent variables, the spatial Durbin model decomposes the impact of an independent variable on the dependent variable into direct, indirect, and total effects. The total effect represents the average impact of an independent variable on the dependent variable across all regions (including both the local and neighboring regions). The direct effect refers to the impact of an independent variable on the dependent variable within the same region. The indirect effect (or spatial spillover effect) captures the influence of an independent variable on the dependent variable in neighboring regions.

The selection and specification of the spatial econometric model in this study were based on a series of statistical tests, with results summarized in Table 1. Initially, both the Lagrange Multiplier (LM) test and the Hausman test were conducted to determine the most appropriate model form. The LM test results indicated the presence of significant spatial autocorrelation across regions, with both spatial error and spatial lag effects being statistically significant (*P* < 0.05). Furthermore, robustness assessments—including the Likelihood Ratio (LR) test and the Wald test—confirmed that the spatial Durbin model is appropriate and does not degenerate into either a spatial lag or a spatial error model (*P* < 0.05). Therefore, the spatial Durbin model was selected for subsequent analysis. Finally, the Hausman test applied to the spatial Durbin model indicated that the fixed-effects specification is more suitable than the random-effects model (*P* < 0.05), supporting the use of a fixed-effects spatial Durbin model in this study.

**Table 1:** Comprehensive density index of maternal and child health care resources in China, 2017–2021.

District	2017	2018	2019	2020	2021
Beijing	0.241	0.245	0.256	0.257	0.267
Tianjin	0.044	0.039	0.044	0.042	0.045
Hebei	0.247	0.264	0.283	0.301	0.314
Shanxi	0.256	0.344	0.283	0.292	0.318
Neimenggu	0.061	0.063	0.069	0.071	0.074
Liaoning	0.019	0.019	0.019	0.019	0.020
Jilin	0.045	0.046	0.046	0.046	0.048
Heilongjiang	0.048	0.049	0.048	0.046	0.046
Shanghai	0.011	0.011	0.010	0.010	0.010
Jiangsu	0.104	0.119	0.126	0.135	0.176
Zhejiang	0.081	0.084	0.103	0.105	0.110
Anhui	0.059	0.062	0.070	0.087	0.095
Fujian	0.061	0.064	0.211	0.074	0.074
Jiangxi	0.092	0.098	0.106	0.118	0.126
Shandong	0.147	0.156	0.168	0.172	0.181
Henan	0.169	0.186	0.198	0.203	0.215
Hubei	0.197	0.205	0.213	0.221	0.233
Hunan	0.116	0.118	0.129	0.127	0.123
Guangdong	0.151	0.161	0.173	0.178	0.187
Guangxi	0.139	0.147	0.157	0.165	0.171
Hainan	0.020	0.022	0.024	0.028	0.030
Chongqing	0.061	0.066	0.070	0.074	0.077
Sichuan	0.066	0.070	0.075	0.079	0.083
Guizhou	0.315	0.353	0.382	0.421	0.486
Yunnan	0.084	0.090	0.258	0.120	0.131
Xizang	0.005	0.003	0.004	0.004	0.005
Shaanxi	0.442	0.458	0.500	0.586	0.521
Gansu	0.021	0.015	0.025	0.027	0.032
Qinghai	0.005	0.006	0.006	0.006	0.006
Ningxia	0.019	0.020	0.022	0.024	0.026
Xinjiang	0.035	0.036	0.038	0.041	0.043
Mean value	0.108	0.117	0.133	0.132	0.138

Spatial Durbin model can better analyze the interaction effects among related variables, and explanatory variables and random error terms are not affected by spatial dependence^36^, and its calculation formula is as follows: $Y = \rho WY + \beta X + \theta WX + \varepsilon$, Y is the dependent variable; X is the independent variable; W is a space matrix; ρ makes the space autoregressive coefficient; β makes regression spatial coefficient; ε represents the random error term. Regional maternal and child health resource allocation efficiency is not only related to local independent variables, but also has a certain impact on its own variables in neighboring regions. Therefore, this study uses the spatial Durbin model to explore the effect of comprehensive maternal and child health resources on efficiency.

This paper adopts the economic distance spatial weighting matrix as the primary spatial weighting matrix. The rationale for this choice is threefold. First, existing studies have shown that the spatial spillover effects of healthcare resource allocation efficiency are driven not only by geographical proximity but also by economic linkages^37^, such as interregional economic cooperation, fiscal transfers, and the flow of healthcare professionals and patients from less developed to more developed regions. Second, provinces with similar levels of economic development exhibit certain similarities in healthcare resource allocation, policy implementation, and the structure of service demand. The economic distance matrix effectively captures such similarity. Third, in a large country like China, geographical distance alone may not fully reflect the intensity of interregional interactions, especially in a context where high-speed transportation networks and digital health infrastructure increasingly facilitate cross-regional exchanges. Thus, the economic distance matrix provides a theoretically and empirically appropriate framework for examining the spatial effects of maternal and child health resource efficiency.

## Results

### Comprehensive maternal and child health resource density index analysis in 31 provinces of China

**Fig. 1:**
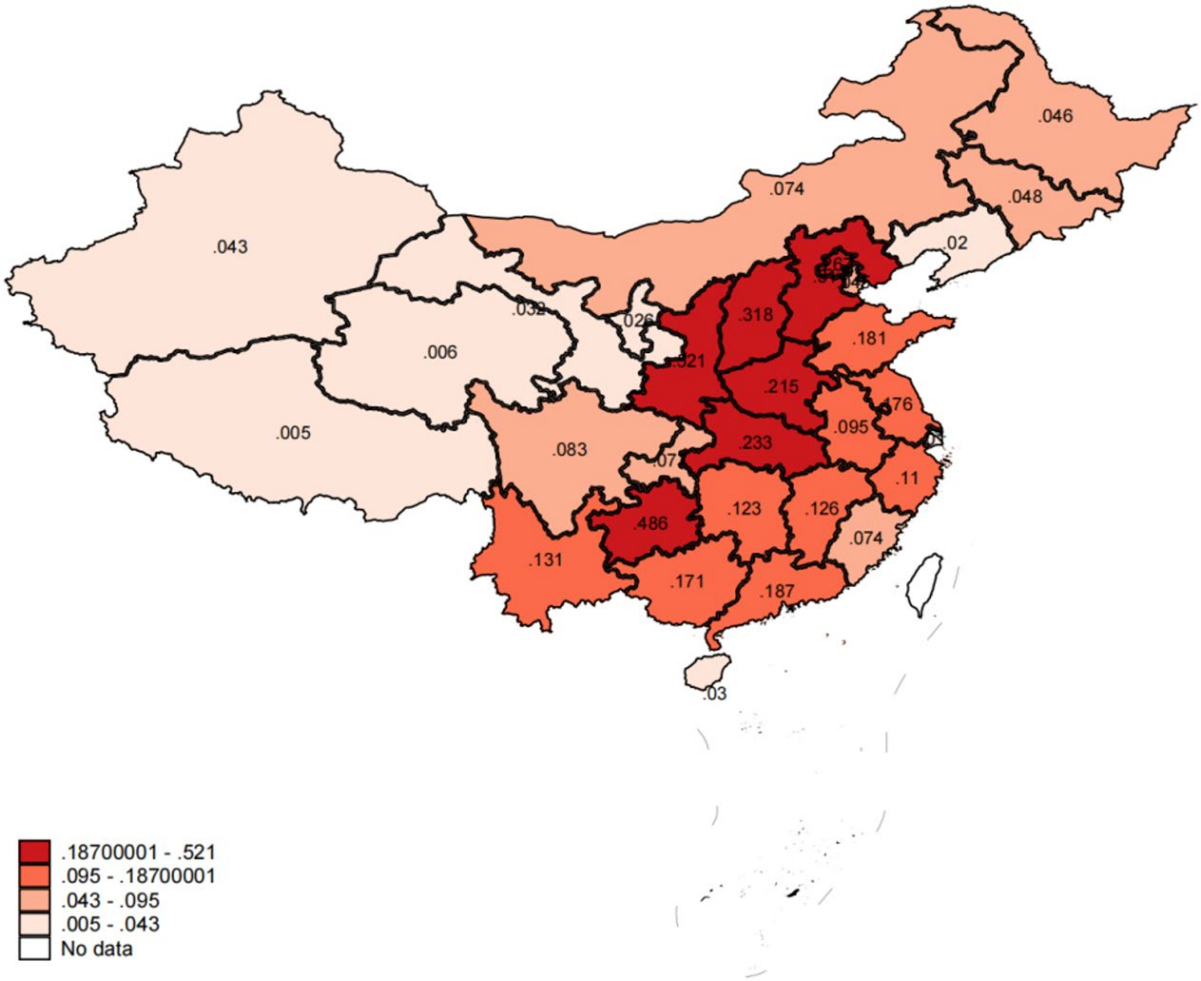
CHRDI maps of different regions.

Based on the health resource density index (HRDI) formula, this study calculated the maternal and child health resource density index (MCHRDI) for China. By integrating the indicator weights derived from the entropy weight method, the comprehensive maternal and child health resource density index for each province in China from 2017 to 2021 was computed, as presented in Table 1. From a temporal perspective, the results reveal an overall upward trend in the comprehensive MCHRDI across all 31 provinces and municipalities, suggesting a gradual improvement in the allocation of maternal and child health resources in China during the study period. Spatial analysis indicates that the comprehensive MCHRDI values of Beijing, Hebei, Shanxi, Hubei, Guizhou, and Shaanxi are significantly higher than those of other provinces. This reflects a relatively higher level of maternal and child health resource allocation in these six provinces and municipalities, as measured by both population and geographical area. The CHRDI maps of different regions drawn using 2021 as an example are shown in Fig. 1.

### Analysis of input–output status of maternal and child health care resources in China

Between 2017 and 2021, China witnessed a decline in the number of maternal and child healthcare institutions. In contrast, the counts of health technicians and hospital beds increased, with the most substantial growth observed in health technicians. Regarding service outputs, the number of live births dropped markedly, decreasing by approximately 60% in 2021 compared to 2017. Conversely, several key health management rates—including those for newborn visits, children under 7 years old, and premarital check-ups—rose. The premarital check-up rate increased the most significantly (by 24%) but was characterized by a low mean and a large standard deviation across provinces, indicating substantial regional disparities in its implementation. (Table 2).

**Table 2:** Input and output of maternal and child health care resources in China from 2017 to 2021 ($\overline{X} \pm \sigma$).

Resource index		2017	2018	2019	2020	2021
input	Number of mechanisms (units)	99 ± 50	99 ± 50	99 ± 50	98 ± 50	98 ± 50
	Number of health technicians (persons)	11,392 ± 9462	12,161 ± 10,208	13,066 ± 11,110	13,833 ± 11,585	14,651 ± 12,033
	Number of beds (sheets)	7098 ± 6491	7511 ± 6789	7848 ± 7037	8161 ± 7042	8393 ± 7249
output	Neonatal visit rate(%)	94.03 ± 3.68	93.63 ± 5.17	93.70 ± 4.86	95.87 ± 1.96	96.54 ± 1.99
	System management rate(%)	89.80 ± 8.40	89.38 ± 7.78	89.66 ± 7.34	92.63 ± 4.18	93.12 ± 4.09
	Premarital examination rate(%)	52.30 ± 31.45	53.32 ± 21.08	54.83 ± 30.16	62.14 ± 27.29	64.62 ± 26.58
	Number of live births (persons)	567,059 ± 462,235	439,402 ± 339,470	469,397 ± 366,346	388,210 ± 307,842	339,203 ± 270,337
	Health care management rate for children under 7 years of age (%)	92.45 ± 4.96	92.48 ± 4.85	93.38 ± 4.39	94.22 ± 2.53	94.57 ± 2.63

### Comprehensive technical efficiency analysis of maternal and child health care resources in China

This study evaluated the resource efficiency of maternal and child health care in China from 2017 to 2021 using a three-stage Data Envelopment Analysis (DEA) model. Table 3 presents both the initial efficiency scores from the traditional DEA model and the adjusted scores after accounting for environmental variables-namely, GDP per capita, population density, and per capita total health expenditure. The results show that incorporating these environmental factors generally led to an increase in technical efficiency, suggesting that, overall, these variables exert a positive influence on resource efficiency. From a temporal perspective, the adjusted technical efficiency scores exhibited a slight declining trend across most regions over the five-year period. Geographically, the bias-corrected analysis revealed striking regional disparities. The highest average efficiency scores, approaching the efficient frontier of 1, were observed in Beijing, Tianjin, Shanghai, Hainan, Qinghai, and Ningxia. In contrast, Hebei and Henan provinces registered the lowest average scores, falling below 0.5.

**Table 3:** Technical efficiency analysis of maternal and child health care resources based on three-stage DEA model.

District	2017		2018		2019		2020		2021		True efficiency mean
	Original efficiency value	True efficiency value	Original efficiency value	True efficiency value	Original efficiency value	True efficiency value	Original efficiency value	True efficiency value	Original efficiency value	True efficiency value	
Beijing	1.000	0.994	0.999	0.997	0.956	0.984	1.000	1.000	1.000	1.000	0.995
Tianjin	1.000	0.999	1.000	0.987	1.000	0.852	1.000	0.982	1.000	0.991	0.962
Hebei	0.097	0.923	0.100	0.888	0.096	0.402	0.109	0.249	0.120	0.873	0.667
Shanxi	0.142	0.928	0.137	0.889	0.136	0.871	0.193	0.377	0.228	0.904	0.794
Neimenggu	0.294	0.949	0.268	0.952	0.224	0.547	0.230	0.538	0.223	0.906	0.778
Liaoning	0.267	0.955	0.249	0.919	0.294	0.842	0.266	0.620	0.261	0.953	0.858
Jilin	0.280	0.974	0.281	0.965	0.268	0.708	0.277	0.750	0.260	0.977	0.875
Heilongjiang	0.160	0.955	0.139	0.910	0.163	0.530	0.157	0.399	0.163	0.951	0.749
Shanghai	0.999	0.999	1.000	1.000	1.000	1.000	0.959	1.000	0.904	1.000	1.000
Jiangsu	0.222	0.818	0.207	0.938	0.206	0.536	0.223	0.514	0.203	0.875	0.736
Zhejiang	0.241	0.814	0.250	0.832	0.254	0.621	0.268	0.483	0.241	0.963	0.743
Anhui	0.338	0.838	0.310	0.881	0.260	0.528	0.262	0.562	0.242	0.791	0.720
Fujian	0.221	0.953	0.218	0.936	0.203	0.609	0.187	0.417	0.176	0.953	0.774
Jiangxi	0.175	0.953	0.192	0.956	0.231	0.554	0.235	0.452	0.227	0.768	0.737
Shandong	0.122	0.940	0.123	0.917	0.137	0.453	0.143	0.293	0.143	0.926	0.706
Henan	0.117	0.896	0.118	0.878	0.129	0.427	0.130	0.271	0.122	0.864	0.667
Hubei	0.193	0.952	0.193	0.941	0.191	0.606	0.177	0.368	0.169	0.945	0.762
Hunan	0.152	0.984	0.166	0.914	0.175	0.516	0.181	0.364	0.176	0.884	0.732
Guangdong	0.151	0.957	0.150	0.930	0.144	0.580	0.135	0.303	0.130	0.933	0.741
Guangxi	0.219	0.839	0.239	0.890	0.255	0.601	0.259	0.491	0.249	0.810	0.726
Hainan	0.763	0.915	0.770	0.920	0.737	1.000	0.788	0.925	0.676	1.000	0.952
Chongqing	0.450	0.943	0.448	0.938	0.449	1.000	0.434	0.670	0.423	0.980	0.906
Sichuan	0.112	1.000	0.119	0.969	0.124	0.628	0.129	0.315	0.127	0.810	0.744
Guizhou	0.188	0.922	0.189	0.916	0.185	1.000	0.177	0.386	0.166	0.948	0.834
Yunnan	0.197	0.969	0.191	0.912	0.148	0.532	0.166	0.328	0.170	0.846	0.717
Xizang	1.000	0.950	1.000	0.731	1.000	1.000	1.000	0.968	1.000	0.860	0.902
Shaanxi	0.169	0.969	0.171	0.954	0.162	0.671	0.155	0.355	0.158	0.952	0.780
Gansu	0.212	0.969	0.197	0.949	0.185	0.813	0.181	0.429	0.168	0.959	0.824
Qinghai	0.852	1.000	0.740	1.000	0.744	1.000	0.979	1.000	1.000	1.000	1.000
Ningxia	1.000	1.000	1.000	1.000	1.000	1.000	1.000	1.000	1.000	1.000	1.000
Xinjiang	0.600	0.980	0.480	0.947	0.435	0.734	0.431	0.816	0.396	0.898	0.875
Mean value	0.385	0.943	0.376	0.928	0.371	0.714	0.382	0.569	0.372	0.920	-

### Spatial econometric analysis of maternal and child health resources in China

#### Spatial Moran index analysis

The global Moran’s I indices for the technical efficiency of maternal and child health resources in China from 2017 to 2021 are presented in Table 4. The results indicate the presence of significant spatial autocorrelation (*P* < 0.05), with Moran’s I values ranging between -0.349 to -0.245. The findings indicate a negative direction of spatial autocorrelation, implying spatial disparities between provinces and cities that share similar levels of efficiency in maternal and child health resource allocation. According to the analysis of Moran’s index, the negative spatial spillover effect of MCH resource allocation efficiency in China is weakening, indicating that the regional MCH resource allocation efficiency in China is developing towards a more equitable trend.

**Table 4:** Global Moran index of technical efficiency of maternal and child health resources in China from 2017 to 2021.

Year	I	E(I)	sd(I)	z	*P*
2017	− 0.348	− 0.033	0.148	− 2.126	0.017
2018	− 0.310	− 0.033	0.148	− 1.870	0.031
2019	− 0.349	− 0.033	0.147	− 2.152	0.016
2020	− 0.245	− 0.033	0.146	− 1.451	0.073
2021	− 0.275	− 0.033	0.148	− 1.632	0.051

The I in the table represents the value of the Moran index, E (I) is the expected value of the Moran Index, sd (I) is the standard deviation of the Moran Index, and z is the standardized value of the Moran Index.

In order to further explore the spatial correlation of maternal and child health care resources among provinces in China, this study conducted a local Moran’s I analysis based on the technical efficiency values of these resources from 2017 to 2021. The data show that Heilongjiang, Inner Mongolia, Liaoning, and Jilin fell into “high-low” or “low–high” agglomeration areas several times during the five-year period, indicating significant regional differences in the development of maternal and child health (MCH) in Northeast China. Regarding the development trend, the number of provinces located in “high-low” or “low–high” agglomeration areas is decreasing, suggesting that China’s maternal and child health care development may be evolving toward a reduction in regional disparities. Provinces not listed in the table exhibited insignificant local Moran’s I results (Table 5).

**Table 5:** Local Moran index of technical efficiency of maternal and child health resources in China from 2017 to 2021.

Year	HH	HL	LL	LH
2017		Shandong	Jilin, Shaanxi	Neimenggu, Heilongjiang
2018				Heilongjiang
2019				Heilongjiang
2020		Liaoning		Heilongjiang
2021	Beijing, Shanghai	Liaoning, Anhui		

HH refers to high-high aggregation area. HL refers to high-low aggregation area, LL refers to low-low aggregation area, LH refers to low–high aggregation area.

#### Spatial Durbin model analysis

In order to explore the impact of maternal and child health care resource density on efficiency in China, this study tested the spatial Durbin model, and the results are shown in Table 6. The fixed-effect SDM coefficient of the independent variable CHRDI is − 0.073 (P < 0.001), indicating that the allocation of MCH health resources has a negative contribution to the service efficiency of MCH. The regression coefficients of population density, the number of institutions, and the number of health technicians are all significantly negative, indicating that an increase in population density and the absolute number of maternal and child health resources is not conducive to the improvement of the efficiency of the regional maternal and child health care system. The regression coefficients of the urbanization rate, total health expenditure per capita, and the number of live births are all positive, indicating that an increase in the urbanization rate and health investment is conducive to the improvement of the efficiency of the regional maternal and child health care system.

**Table 6:** Spatial Durbin model and its effect decomposition.

Variable	Fixed effect SDM	Fixed effect SDM decomposition		
		Direct effect	Indirect effect	Total effect
CHRDI	− 0.073***	− 0.062***	− 0.270***	− 0.331***
Rate of urbanization	0.172*	0.190**	− 0.478**	− 0.287**
Density of population	− 0.018***	− 0.016***	− 0.023	− 0.040**
Total health expenditure per capita	0.041*	0.049*	− 0.213***	− 0.163**
Number of mechanisms	− 0.215***	− 0.216***	0.030	− 0.186***
Number of health technicians	− 0.200***	− 0.215***	0.369***	0.153*
Number of live births	0.380***	0.390***	− 0.259***	0.131*
Wx CHRDI	− 0.334***			
Wx Rate of urbanization	− 0.538**			
Wx Density of population	− 0.032			
Wx Total health expenditure per capita	− 0.236***			
Wx Number of mechanisms	− 0.021			
Wx Number of health technicians	0.390***			
Wx Number of live births	− 0.218*			
rho	− 0.233			
σ²ₑ	0.005			
R^2^	0.786			
Log− likelihood	186.980			
N	155			

The variable coefficients marked ***, ** and * indicate significance at the levels of 99%, 95% and 90%, respectively, and Wx is the spatial lag term of the spatial independent variable.

At the same time, in order to accurately judge the spatial spillover effect of each variable on the resource efficiency of maternal and child health care, this study further decomposes the regression results of the spatial Durbin model and divides the model into direct effect, indirect effect (spatial spillover effect), and total effect. It is found that the direct effect, indirect effect, and total effect of the comprehensive maternal and child health resource density index and population density are significantly negative, indicating that simply increasing the density of health resources and population density will not improve efficiency. The direct effect of the urbanization rate and total health expenditure per capita is significantly positive, while the indirect effect is significant but the coefficient is negative, indicating that an increase in total health expenditure per capita and the urbanization rate has a positive effect on the efficiency of the local region but has a slight inhibitory effect on surrounding regions.

## Discussion

The analysis reveals two contrasting trends in China’s maternal and child health (MCH) sector: while the health resource density index indicates a continuous increase in the quantity of MCH resources, the three-stage DEA model reveals a declining trend in resource efficiency. Both resource allocation and efficiency demonstrate pronounced regional disparities. Furthermore, the global Moran’s Index confirms strengthening spatial autocorrelation in resource efficiency over the study period. Complementing this finding, the spatial Durbin model estimates a significant negative direct effect of local MCH resource density on its own efficiency, suggesting that increased resource concentration may inhibit local performance despite the overall spatial interdependence.

### The resource efficiency of maternal and child health care in China is reduced, and there are highlands and depressions

In this study, the bias-corrected efficiency values obtained by incorporating environmental variables via the stochastic frontier approach account for the influence of external factors, thereby providing a more realistic estimation of the technical efficiency of maternal and child health (MCH) resources. The results indicate a declining trend in the resource efficiency of MCH care in China over time, alongside significant regional disparities in the geographical dimension, characterised by the coexistence of “efficiency highlands” and “efficiency depressions”. These findings are consistent with prior research by Leng et al.^38^. Moreover, the introduced economic, social, and demographic factors are shown to exert considerable influence on MCH resource efficiency. This suggests that the Chinese government should conduct an in-depth analysis of how the external environment affects MCH efficiency.

Additionally, this study observed a decreasing trend in the number of MCH institutions in most regions of China. Some studies suggest that excessive health resource inputs may lead to diminishing returns under certain institutional conditions^39^. Additionally, demographic research has noted that improved social welfare and elderly support may correlate with reduced fertility intentions in some contexts^40^, which could partially explain reduced demand for MCH services. At the same time, adjustments in maternity policy have raised the average age of pregnant women and increased expectations for the quality of MCH care. Under these circumstances, simply reducing or closing underperforming institutions is not an appropriate strategy to mitigate resource waste. Instead, this study emphasises the importance of enhancing the management capacity of professional healthcare institutions. We propose improving the performance evaluation system for MCH institutions in China, promoting and incentivising management innovation, fostering a supportive working environment for MCH staff, and elevating service quality to reduce talent attrition and equipment idleness in grassroots institutions^41^. Concurrently, it is essential to increase financial investment and strengthen the managerial capacity of professional health institutions to mitigate the syphoning effect of talent by large hospitals.

### The density of maternal and child health care resources in China has increased, and the regional differences are large

The health resource density index (HRDI) employed in this study incorporates both population and geographical dimensions, thereby providing a comprehensive assessment that reflects both the equity and accessibility of medical resource distribution. Consequently, the estimated allocation level of maternal and child health (MCH) resources derived from this index more accurately approximates the actual situation. The results reveal a general increase in the density of MCH resource allocation across China over time, while also highlighting significant regional disparities and a pronounced polarisation in the spatial dimension. These findings align with previous research by Xuhui W et al.^42^, indicating that a higher number of provinces in Northwest and Northeast China exhibit relatively poor resource allocation levels. This regional imbalance may be attributed to uneven economic development^43^. Many underdeveloped provinces are characterised by net population outflow, and their geographical features—such as mountainous terrain or vast land area—contribute to low population density^44^. As a result, MCH resources in these regions are often dispersed, leading to a lower resource density allocation level.

To address these disparities, we recommend that less developed regions strengthen governmental responsibility and enhance the operational and management mechanisms of relevant policies at the institutional level. Macro-level policies should encourage and guide each region to implement tailored strategies according to local conditions. Furthermore, greater attention should be paid to attracting skilled professionals through improved mechanisms for recruitment, training, and compensation. It is also essential to establish robust assessment and supervision systems for professional health institutions. These measures are expected to elevate the input level and service quality of MCH care, particularly in disadvantaged areas such as the Northeast and Northwest. By providing efficient and targeted support, policymakers can improve the fairness and balance of professional medical services, narrow regional gaps, and ultimately ensure that all residents have equitable access to MCH resources.

### The efficiency of maternal and child health care resources in China has spatial autocorrelation, and it shows an increasing trend

In this study, the Moran Index was used to analyze the spatial distribution of the efficiency of maternal and child health resources in China. China’s maternal and child health care resources may be concentrated in a few advantageous areas, forming a “siphon effect”, which makes the resources in the surrounding areas relatively insufficient or the utilization efficiency decreased. This conclusion is consistent with that of scholar Qin Q^7^. Therefore, this study suggests that the central government should pay timely attention to the cold hot spots of maternal and child health resource efficiency at the macro level, and encourage inter-regional exchanges and cooperation activities to share resources, technology and experience, and promote the effective expansion of high-quality resources through technical assistance, personnel training and other means, such as: Neighboring regions will carry out experience sharing meetings on the relief of major maternal and child diseases and encourage multi-party consultation activities, so as to achieve the purpose of promoting the common improvement of the allocation level of maternal and child health resources in the region, so as to form a virtuous cycle mechanism of high level driving low level common development.

### The increase of maternal and child health resource density in China has a spatial spillover effect

In this study, the spatial Durbin model was employed to examine the spatial spillover effects of maternal and child health care (MCH) efficiency in China, effectively accounting for potential biases arising from spatial dependence^45,46^. The results show that although the level of maternal and child health care resource density in China gradually increased from 2017 to 2021, the efficiency of maternal and child health care institutions showed a slight downward trend. This finding is consistent with the conclusion proposed by Sun X et al.^19^ that the resource density of the primary health care system affects its efficiency. In light of this seemingly paradoxical phenomenon—”increasing resource density while decreasing efficiency”—this paper argues that the underlying causes are multifaceted, resulting from the combined effects of declining fertility rates (leading to demand-side contraction), a mismatch between resource allocation and demand structure, and insufficient internal management efficiency. First, from the perspective of supply–demand balance, the continuous decline in fertility rates in recent years has led to weak or even negative growth in actual demand for maternal and child health care services. Meanwhile, increased resource density has created relative oversupply, intensified regional competition among institutions, and caused some institutions to experience “underfeeding,” thereby reducing overall technical efficiency. Second, an optimal match between resource allocation, population distribution, and service demand structure has not yet been achieved. In some regions, resources are concentrated in specific types or levels of institutions, while the allocation of personnel, equipment, and service capacity in grassroots maternal and child health institutions remains relatively weak, leading to idle or inefficient resource use. Third, low management efficiency further limits the ability to convert resources into outputs. Some institutions still have management deficiencies in service processes, performance assessment, and information systems, making it difficult to adapt to the requirements of refined management following resource expansion. Moreover, from the perspective of patients’ subjective demand for maternal and child health services, patients are often influenced by hospital hierarchy and prefer large hospitals with strong comprehensive capabilities, which further compresses the service volume of grassroots maternal and child health institutions^47^. The key lies in optimizing the regional resource allocation structure, enhancing institutional management capacity, and aligning these efforts with realistic conditions such as changes in fertility rates and residents’ medical preferences.

Based on these findings, this study recommends that MCH policy formulation take into account regional external environmental factors, including socioeconomic conditions, residents’ medical needs, affordability, accessibility, and service quality. Tailored local policies and optimized operational mechanisms are essential to improve efficiency and encourage the use of specialized MCH services. Moreover, rather than indiscriminately increasing investments or institution density, resources should be allocated rationally, with emphasis on enhancing service efficiency and quality. Care should also be taken to avoid resource siphoning from surrounding areas^48^.

### Urbanization level, population density and per capita total health cost affect the efficiency of maternal and child health care

The increase in the absolute number of maternal and child health (MCH) resources and population density is not conducive to improving the efficiency of regional MCH healthcare systems. Through analyzing the underlying reasons, this study concludes that there are issues in the equity of resource allocation in maternal and child health institutions in China, particularly the most uneven distribution by geographical area. This implies that even if the absolute amount of MCH resources is increasing, the growth is not evenly distributed across regions—especially in less economically developed areas—which may lead to inefficient resource utilization and thus affect overall MCH system efficiency. Meanwhile, an increase in population density does not necessarily harm the efficiency of maternal and child healthcare. However, under conditions such as insufficient supply elasticity, unbalanced resource distribution, and a concentration of high-risk groups, higher density can lead to diminishing marginal efficiency or even negative growth after crossing a certain threshold. The solution lies not in reducing density, but in establishing resource planning that dynamically aligns with density, promoting hierarchical diagnosis and treatment and community-based MCH services, and developing digital follow-up tools to replace part of in-home services.

The increase in urbanization rate, total health expenditure per capita, and the number of live births is conducive to the improvement of regional maternal and child health care system efficiency. This conclusion is consistent with the research findings of Raghupathi V and other scholars^49^. First, urbanization promotes population concentration and infrastructure development, shortening the physical and informational distance of maternal and child health care services. This facilitates the establishment of hierarchical diagnosis, treatment, and referral networks, improves service accessibility and continuity, and thereby enhances the overall operational efficiency of the system. Second, the increase in total health expenditure per capita directly reflects greater health investment by both the government and society. This increase provides more adequate financial security for maternal and child health care, enabling improvements in service facilities, personnel training, and the establishment of performance incentive mechanisms. It also reduces the economic barriers to seeking medical care for families, thereby promoting dual improvements in resource utilization efficiency and service quality. Finally, under certain resource conditions, a moderate increase in the number of live births can generate economies of scale in service delivery, dilute fixed costs, encourage medical staff to accumulate clinical experience, and provide a sufficient data basis for monitoring and improvement. Consequently, this enhances the overall efficiency of the service process. Taken together, these factors collectively promote the optimization of regional maternal and child health care system efficiency.

Increases in per capita total health expenditure exert a positive effect on local maternal and child health (MCH) efficiency, while generating a modest inhibitory effect on surrounding regions. This study suggests that such increases reflect enhanced regional investment in MCH, which can produce a resource agglomeration effect, thereby improving the efficiency of local MCH service delivery. However, while local efficiency gains are achieved, the concentration of resources may mildly suppress efficiency in neighbouring areas. This could be attributed to cross-regional resource competition, leading to relative resource depletion in adjacent regions, or to the outflow of skilled health workers toward more resource-abundant areas. Furthermore, although the technical efficiency of health resource allocation in China has continued to improve, returns to scale have shown a trend toward stabilisation^50^. The accumulation of health resources in one region may enhance its technical efficiency, whereas surrounding regions-facing fragmented resources and insufficient scale—may fail to achieve economies of scale, thus constraining their efficiency levels.

### International comparisons and regional generalizability of findings

The findings of this study not only hold policy implications for China but also, through comparative analysis with relevant international literature, reveal the common challenges and differentiated pathways in resource allocation and the efficiency of maternal and child health care worldwide. Methodologically, the combination of the entropy weight method and the DEA model used in this study has been widely validated by European scholars. Grausova and Hužvar applied the entropy weight-DEA model to assess the efficiency of European medical systems, confirming the rationality of the entropy weight method in evaluating resource allocation^51^. Similarly, the study by Dziechciarz and Szczeciński further verified the applicability of the DEA model in analyzing the performance of health systems in EU countries^52^. The comprehensive health resource density index (CHRDI) constructed in this study aligns with the multidimensional evaluation frameworks adopted in European studies, as both emphasize the dual consideration of demographic and geographical dimensions. In comparisons with the Middle East and transition economies, the Malmquist index analysis of public hospitals in Azerbaijan reveals that productivity changes are primarily driven by technological progress or shifts in management efficiency and suggests improving efficiency through technological inputs^53^. This finding resonates with the emphasis of this study on management capacity building and performance evaluation system optimization, forming a cross-regional consensus. Selamzade’s study on G20 countries further underscores the central role of the health workforce in addressing global challenges and recommends increasing the number of health professionals while optimizing their distribution^54,55^. In comparisons with Africa and other developing countries, although China, as a middle-income country, is at a different stage of development from African nations, the study by Selamzade and Yeşilyurt provides an important reference for research on health system efficiency in African countries^56^. This study finds that improving the accessibility and quality of medical care can significantly enhance system efficiency, which is highly consistent with the recommendation of “optimizing resource allocation structure and improving service quality” proposed in this study. This consistency serves as a warning that China needs to strengthen governance mechanisms alongside resource expansion. Regarding the international applicability of the policy recommendations, although the “coordination strategy” proposed in this study is grounded in the Chinese context, its core logic remains open to dialogue with different health system models. Compared with the social insurance-based European system, China’s public-sector-led model places greater emphasis on direct government input and administrative coordination. In contrast to the public-sector-led model in the Middle East, China’s regional collaboration mechanisms (e.g., the Beijing-Tianjin-Hebei coordinated development) demonstrate unique spatial governance innovations. Research on Brazilian public hospitals suggests that “lean medicine” can translate into efficiency gains^57^, which aligns with the direction of management refinement emphasized in this study. A DEA study of the prison system in Minas Gerais state recommends the adoption of differentiated management strategies^58^, which also supports the policy propositions of this study regarding localized and category-based guidance. Finally, a study by Selamzade et al., focusing on the COVID-19 period in OECD countries, emphasizes that rapid policy intervention is crucial for inefficient countries^59^. This echoes the policy coordination strategy proposed in this study, indicating that optimal resource allocation requires timely institutional responses.

### Limitations

It should be noted that the analysis of health resource allocation based on population dimension is limited to the permanent population and does not consider the floating population, which may affect the generalisability of the results.

The ability to establish causality in this study is relatively weak. Although the spatial Durbin model is employed and lagged terms of variables are introduced to partially mitigate endogeneity issues such as reverse causality, the model essentially still captures correlations between variables. It remains difficult to completely rule out the influence of omitted variables and reverse causality—for example, regions with abundant medical resources may have adopted digital economy initiatives earlier or possess stronger financial capacity, thereby simultaneously improving resource allocation and system efficiency. Therefore, the findings of this study should be interpreted as correlational rather than strictly causal. Additionally, certain indicators that are difficult to quantify or inaccessible through public data—such as the degree of policy implementation, institutional execution capacity, and institutional wage levels—are not included in the analysis of influencing factors. These variables may nevertheless affect the efficiency of maternal and child health systems. Future research could more robustly identify the causal effects of maternal and child health (MCH) resource allocation on efficiency by collecting data over a longer time span, introducing appropriate instrumental variables, or employing quasi-natural experiments.

In terms of data sources, this study primarily relies on secondary statistical yearbook data. Although this type of data is highly authoritative and accessible, it may still be subject to reporting bias. Regarding institutional coverage, the study is constrained by data availability. Only specialized maternal and child health (MCH) institutions are included; resources and services provided by private MCH institutions, general hospitals, and maternity hospitals are not incorporated into the analysis. While the latter account for a relatively small share overall, they may still play a role in certain regions or specific services. Their exclusion could introduce subtle biases in understanding resource allocation patterns and service efficiency. As for confounding factor control, due to data limitations, this study fails to account for several unobserved factors that may influence both resource allocation and institutional efficiency. These include regional cultural norms (e.g., preferences regarding the utilization of maternal and child health services), local governance quality (e.g., government willingness to invest in health and capacity for policy implementation), and the broader socioeconomic environment. The presence of these factors may affect the study’s conclusions. Future research will aim to expand data sources by incorporating maternal and child health resources and services provided by general hospitals and maternity hospitals. At the same time, panel data models and other methods will be employed to more accurately identify the causal relationship between resource allocation and efficiency in maternal and child health care. In addition, AI-related indicators were not included in this study. Future research could consider incorporating emerging technological indicators, such as smart medical care and remote maternal and child health services.

The index system of primary medical resources constructed in this study is primarily based on macro-level statistical data and indicators derived from health annual reports. The selected indicators mainly capture changes in resource input and service volume (e.g., number of institutions, number of beds, personnel allocation, total financial subsidies, and number of patient visits), but they fall short of fully incorporating quality-related dimensions that are directly associated with service quality, patient experience, and health outcomes (e.g., diagnostic and treatment standardization, patient satisfaction, chronic disease control rate, and level of health improvement). This limitation may hinder the system’s ability to comprehensively assess the “actual effectiveness” and “public sense of gain” of primary healthcare services. Moreover, it reduces the explanatory power of the research findings in guiding quality enhancement and performance improvement. Future research will focus on integrating individual-level health data and service process data at the micro level. By leveraging multi-source data platforms—such as electronic health records, patient follow-up surveys, and clinical quality monitoring systems—patient-centered quality evaluation indicators will be progressively incorporated. Ultimately, this will facilitate the development of a comprehensive evaluation system for primary medical resources that equally emphasizes quantity and quality, as well as structure and outcomes.

The current output indicators have some limitations, such as being unable to distinguish the difference in resource consumption between normal and high-risk delivery, and difficult to reflect the quality connotation in the service process. Therefore, future research should integrate more refined service output data as much as possible, such as the number of high-risk pregnant women under management and the number of obstetric complications treated, so as to further improve the accuracy of evaluation and the explanatory power of policy.

## Conclusion

The level and efficiency of health resource allocation represent a central concern for health policy makers and administrators. This study presents an empirical analysis of the allocation and efficiency of medical resources within China’s maternal and child health (MCH) system and examines the relationship between them. Our findings indicate that between 2017 and 2021, although MCH resource density increased in most regions of China, technical efficiency generally declined, and interregional disparities in resource allocation widened. Only a few regions achieved an efficient state. We further identify a spatial spillover effect of MCH resource allocation on efficiency, with the comprehensive CHRDI in maternal and child health, urbanisation rate, and total health expenditure per capita as key factors influencing technical efficiency. To address these issues, we recommend that China optimise its MCH resource allocation through coordinated policy guidance, financial support, performance evaluation, and regional collaborative development, with the ultimate goal of improving system-wide efficiency.

## Data Availability

The datasets generated and analyzed during the current study are available in the Zenodo repository, [https://zenodo.org/records/20048870].

## Abbreviations

HRDI: Health resource density index
CHRDI: Comprehensive health resource density index
HH: High-high
LH: Low-high
LL: Low-low
HL: High-low
